# Factors affecting utilization of skilled maternal care in Northwest Ethiopia: a multilevel analysis

**DOI:** 10.1186/1472-698X-13-20

**Published:** 2013-04-15

**Authors:** Abebaw Gebeyehu Worku, Alemayehu Worku Yalew, Mesganaw Fantahun Afework

**Affiliations:** 1 Department of Reproductive Health, Institute of Public Health, University of Gondar, Gondar, Ethiopia; 2 Department of Preventive Medicine, School of Public Health, Addis Ababa University, Addis Ababa, Ethiopia; 3 Department of Reproductive Health and Health Service Management, School of Public Health, Addis Ababa University, Addis Ababa, Ethiopia

**Keywords:** Skilled maternal care, Linked survey, Multilevel analysis

## Abstract

**Background:**

The evaluation of all potential sources of low skilled maternal care utilization is crucial for Ethiopia. Previous studies have largely disregarded the contribution of different levels. This study was planned to assess the effect of individual, communal, and health facility characteristics in the utilization of antenatal, delivery, and postnatal care by a skilled provider.

**Methods:**

A linked facility and population-based survey was conducted over three months (January - March 2012) in twelve “kebeles” of North Gondar Zone, Amhara Region. A total of 1668 women who had births in the year preceding the survey were selected for analysis. Using a multilevel modelling, we examined the effect of cluster variation and a number of individual, communal (kebele), and facility-related variables for skilled maternal care utilization.

**Result:**

About 32.3%, 13.8% and 6.3% of the women had the chance to get skilled providers for their antenatal, delivery and postnatal care, respectively. A significant heterogeneity was observed among clusters for each indicator of skilled maternal care utilization. At the individual level, variables related to awareness and perceptions were found to be much more relevant for skilled maternal service utilization. Preference for skilled providers and previous experience of antenatal care were consistently strong predictors of all indicators of skilled maternal health care utilizations. Birth order, maternal education, and awareness about health facilities to get skilled professionals were consistently strong predictors of skilled antenatal and delivery care use. Communal factors were relevant for both delivery and postnatal care, whereas the characteristics of a health facility were more relevant for use of skilled delivery care than other maternity services.

**Conclusion:**

Factors operating at individual and “kebele” levels play a significant role in determining utilization of skilled maternal health services. Interventions to create better community awareness and perception about skilled providers and their care, and ensuring the seamless performance of health care facilities have been considered crucial to improve skilled maternal services in the study area. Such interventions should target underprivileged women.

## Background

In Ethiopia, maternal health has received attention for the last two decades. Currently, reducing maternal mortality is one of the goals of the Health Service Development Program of the country. Maternal and newborn health is also among the six priority areas in the reproductive health strategy [1]. Based on ecological and historical evidences observed in European and Asian countries, health professionals with midwifery skill were considered the key factors to decrease maternal mortality. The low maternal mortality rates reported by the Netherlands, Norway, and Sweden in the early 20th century were believed to have been the result of an extensive collaboration between physicians and highly competent, locally available midwives [2]. Similarly, Malaysia, Sri Lanka and Thailand halved their maternal mortality ratios within ten years by increasing the number of midwives in the 1950s and 1960s [3].

At the moment, many countries including Ethiopia are working to meet the WHO recommendation of having skilled attendance for all births [4]. According to the recent 2011 Ethiopian Demographic and Health Survey (EDHS) report, only 34%, 10% and 6% of the women had Antenatal Care (ANC), delivery, and postnatal care by skilled providers respectively [5]. Maternal mortality has also not changed from its previous level where maternal mortality ratio was 673 per 100,000 live births in the 2005 EDHS report [6], and it was 676 per 100,000 live births in the 2011 EDHS report [5].

Skilled maternal care refers to maternity services (antenatal, delivery, and postnatal care) by a health professional with midwifery skill that can be provided at different levels (home, health centers or hospitals). In order to provide such skilled maternal care, we need to have an enabling environment and skilled providers. An enabling environment include; functional health facilities and a reliable referral system to link the different levels, awareness and readiness of the community for utilizing skilled care as well as supporting the policy and political commitment [4]. Health professionals who have been educated and trained to proficiency in the skills needed to manage normal pregnancies, childbirth and the immediate postnatal period, and in the identification, management or referral of complications are categorized as skilled care providers [7,8]. In the study, skilled providers include midwives, nurses, health officers and doctors.

There are several factors influencing skilled maternal health care utilization within the dimensions of skilled maternal care definition. The factors operate their impact at different levels. Review of the global literature indicates that these factors can be classified as 1) individual and household level 2) communal and health facility level and 3) national or state level.

At the individual and household level maternal education, parity, residence, awareness and perceptions related to the risks of pregnancy and skilled maternal services, previous experiences, women’s decision-making power, and household wealth are some of the known variables.

At the health facility level the availability, readiness, and quality of services as well as the type, competence and caring behavior of providers are very important for maternal services. However, in many developing countries health facilities are not performing the expected functions according to their level [9]. Furthermore, there is a huge gap in the competence of health workers categorized as skilled providers [8,10]. Pre-service training is also not a guarantee. It is recommended that maternity care providers need to receive refresher training or updates in midwifery every three to five years [11].

Some studies identified community and state level factors using multilevel analysis. A study in Vietnam reported that the presence of low education coverage, geographical isolation, and high poverty rate in the community reduce access to skilled maternal services [12]. Evidences from Nigeria also showed that urban residence and the availability of community media significantly increased maternal service utilization [13]. At the policy level, resource investments in the main system components of health programs raised the level of maternal service utilizations [14]. The significant effect of government health expenditure in improving the utilization of skilled maternal service was also observed in other studies [15,16].

Studies in Ethiopia have serious limitations in evaluating the determinants of skilled maternal care utilization. The studies provided evidence based on the evaluation of either user or facility related determinants. Addressing only one of these categories is likely to present a very incomplete picture of the evaluation of determinants affecting maternal service utilization. In addition, the nesting effects of different levels are usually not controlled during analysis. Therefore, appropriate methodology is required for a more comprehensive and accurate analysis. As a result, all potential sources of poor skilled maternal care utilization can be evaluated to design maternal service strategies at different levels. This study was planned to assess the effect of individual, household, communal and health facility characteristics in the utilization of antenatal, delivery, and postnatal care by a skilled provider.

## Methods

### Study design and area

A linked facility and population-based survey i.e. a facility survey linked to population based survey sample areas was conducted over three months (January - March 2012) in North Gondar Zone. The two surveys were conducted concurrently to analyze all potential factors operating at different levels (individual and community levels) simultaneously. The use of linked survey is also considered as stronger approach to analyze causality in non-experimental studies [17].

North Gondar is one of the 11 zones of the Amhara Regional State located in northwest Ethiopia. It has 22 districts. The zonal center, Gondar town, is located 735 kilometer from Addis Ababa. At the time of the study, more than three million individuals were living in the zone. About 84.1% of its inhabitants were rural dwellers [18]. North Gondar, the largest zone of the region, is known for its difficult topography.

### Study population and sampling

Women who had births in the year preceding the survey were included in the study. A multistage sampling technique was applied to select the study population. Initially, six districts of the zone namely Dembia, Lay Armachiho, West Belessa, Metema, Debark and Dabat were selected. Then a total of twelve clusters or kebeles (two kebeles per district) were selected randomly. The ‘kebeles’ are the lowest administrative units with a size of about 1000 households (5000 population). All eligible women found in the selected cluster were studied. Accordingly, 1730 women were identified for interview.

The adequacy of the sample size was checked based on differences in frequency of important exposure variables among users and non-users at the time of proposal development. Epi Info StatCalc software was used for the calculations by assuming a 95% confidence level and 80% power with a 1:3 ratio of users and non-users.

In order to have a linked data, basic essential obstetric care facilities (health centers) utilized by the kebele population were selected for the facility based survey. For each cluster (kebele), one basic essential obstetric care facility was studied and linked.

### Data collection

All health facilities serving the study population were assessed with regard to the number, training and competency of obstetric staff; services offered; signal functions; physical infrastructure; and availability, adequacy and functional status of supplies and other essential equipment for maternal services. The basic signal functions include parenteral antibiotics, anticonvulsants and oxytocics, and the procedures for manual removal of the placenta, removal of retained uterine products, and assisted vaginal delivery. During the population based survey information about kebele (cluster) characteristics, socio-demographic characteristics, awareness, perceptions and experiences related to the women’s use of skilled maternal care services were collected.

For the population based survey, a total of 36 qualified data collectors and supervisors (2 data collectors and 1 supervisor for each kebele) were trained and deployed. The interviews were conducted using the local language (Amharic) after the questionnaire was pretested for cultural appropriateness and clarity. For the facility-based survey, 14 health professionals who had work experience were recruited.

### Data analysis and modeling

After appropriate coding, the data were entered using Epi Info version 3.5.3 software and exported to STATA version 11 software for analysis. Uses of maternal services (antenatal, delivery and postnatal care) by skilled providers were the dependent variables. The predictors of each of these indicators were assessed separately using a multilevel binary logistic regression.

The rationales for using a multilevel modeling were the following. Firstly, the utilization patterns of maternal health services are influenced by the characteristics of different levels (individuals, community, and health facilities). Analyzing variables from different levels at one single common level using the standard binary logistic regression model leads to bias (loss of power or Type I error). This approach also suffers from a problem of analysis at the inappropriate level (atomistic or ecological fallacy). Multilevel models allow us to consider the individual level and the group level in the same analysis, rather than having to choose one or the other. Secondly, due to the multistage cluster sampling procedure, individual women were nested within kebeles; hence, the likelihood of women seeking maternal health services is likely to be correlated to the kebele members. The assumption of independence among individuals within the same cluster and the assumption of equal variance across clusters are violated in the case of nested data. Hence, the multilevel analysis is the appropriate method for such cases.

Using a two-level binary logistic regression modelling, we examined the effect of a number of individual, community, and facility variables. During analysis, the characteristics of women and household were taken as individual level (level-1), and characteristics of the kebeles (including characteristics of their health centers) were treated as level-2 (Table 1). For each of the three dependent variables, we estimated two models: intercept-only model; an empty model that contains no covariates, and a full model that included individual and kebele level variables. The full model was a random slopes model for ANC and a random intercept model for delivery and postnatal care.

**Table 1:** Description and measurement of variables included in the models, North Gondar, 2012

**Variables**	**Descriptions**	**Measurements**
**Dependent variables**	Women who received ANC at least once from a skilled provider, delivery attended by a skilled provider and Women who received PNC at least once from a skilled provider	For each indicator, ‘Yes’ responses are coded as ‘1’ otherwise as ‘0’
ANC, Birth attendance and PNC by a skilled provider (three dependent variables)		
**Level-1 predictor variables**	**Individual and household characteristics**	
Birth order	Birth order of women’s most recent birth (index birth)	It is divided in to four categories: first birth, 2nd- 3rd, 4th-5th and above 5th
Maternal and husband educational status	Highest level of education attained by the respondent (mother) and her husband	For each variable, it is categorized in to three: illiterate, primary and secondary and above secondary
Wealth quintile	Household wealth status is computed by principal component analysis from ten variables (presence of own farmland, own toilet facility, bank account, mobile phone, electricity, roof of house with corrugated iron sheet, number of cows/oxen, horses/ mules/donkeys goats/sheep and chicken)	The wealth status is categorized in to five groups and ranked from poorest to wealthiest quintile.
Awareness on risk of pregnancy	Awareness of women on risk of pregnancy	When women mentioned at least one major risk factor, the value is ‘1’ if not it is ‘0’
Health professionals preferred by women	Women prefer health professionals for their maternity care	Yes’ responses are coded as ‘1’ and no responses as ‘0’.
Awareness on places to get skilled providers	Women know appropriate health facilities to get skilled providers	When appropriate facilities are known, the value is ‘1’ if not it is ‘0’.
Pregnancy wontedness	Pregnancy wanted or not	Pregnancy wanted or miss-timed coded as ‘0’ and unwanted coded as ‘1’
ANC in previous pregnancy	Presence of at least one ANC visit in previous pregnancies	Yes’ responses are coded as ‘1’ and no responses as ‘0’.
Agreed on postnatal check up	Women agreed on the need of postnatal check up after normal delivery	When women agreed, the value is ‘1’ if not it is ‘0’.
**Level-2 predictor variables**	**Communal (kebele) characteristics**	
Source of income	The kebele population main source of income	It is categorized in to two: Farming and mixed (farming and trading)
Self sustained	Presence of enough production for feeding the population	Yes’ responses are coded as ‘1’ and no responses as ‘0’.
Average distance	Average distance (in Kilometer) of villages in each kebele to the nearest health center	Supervisors estimated the distances of villages from health centers. The average values were calculated and rounded to one decimal place.
	**Health facility characteristics**	
Presence of signal functions	Presence of all the six signal functions in basic essential obstetric care facilities (health centers)	If all signal functions are present, the value is labeled as ‘1’ if one or more are missing, the value is labeled as ‘0’
Duty service	Maternity service for 24 hrs and 7 days	Yes’ responses are coded as ‘1’ and no responses as ‘0’.
Availability of obstetric guidelines	Obstetric guidelines available for reference by staff at all times	Yes’ responses are coded as ‘1’ and no responses as ‘0’.
Payment requirements	Maternity clients required to purchase/ provide supplies/drugs at time of delivery	Yes’ responses are coded as ‘1’ and no responses as ‘0’.
Overall infrastructure	Infrastructures of the health facility (examination and delivery rooms, refrigerator, water source, power supply, toilet facilities, drainage system…)	Based on records of observation, the infrastructure of the health facility is categorized as not satisfactory (1) and satisfactory (2).
Basic equipments	Presence of basic equipments (BP apparatus, stethoscope, fetal stethoscope, adult and baby weighing scales, delivery and episiotomy sets, sterilizers…)	Based on records of observation, the variable is categorized as not satisfactory (1) and satisfactory (2).

Assume the binary responses Y_ij_ depend on individual-level explanatory variable *X*_*ij*_ and group-level explanatory variable *Z*_*j.*_ If deviation from the average intercept and slope due to cluster (level-2) effect are represented by ***u***_**0*j***_ and ***u***_**1*j***_, the two models are given in the following way.

The intercept-only model = **Logit** *p*_*ij*_ = *γ*_00_ + *u*_0*j*_  and the full model = **Logit**(*p*_*ij*_)  = *γ*_00_ + *γ*_01_ *Z*_*j*_ + *γ*_10_ *X*_*ij*_ + *u*_0*j*_ + *u*_1*j*_ *X*_*ij*_. The intercept **γ**_**00**_ and slopes **γ**_**01**_ and **γ**_**10**_ are fixed effects whereas ***u***_***oj***_ and ***u***_**1*j***_ are random effects of level-2.

The intercept-only model allows us to evaluate the extent of the cluster variation influencing maternal service utilization of women. The intra-class correlation coefficient (Rho) was calculated to evaluate whether the variation in the scores is primarily within or between clusters. In logistic distribution, level-1 residual variance, ε_ij,_ is standardized and fixed with a mean of zero and variance = π^2^/3. Therefore, for a two-level logistic random intercept model with an intercept variance of σ^2^_u0_, the intra-class correlation coefficient (Rho) is given by $‘\rho =\frac{\sigma^{2}{}_{\text{u}0}}{\sigma^{2\;}{}_{\text{u}0}+\pi^{2}/3}’$.

### Ethical considerations

Before the commencement of the study, ethical clearance was obtained from the Institutional Review Board of the College of Health Sciences, Addis Ababa University. Then permission letters from officials of districts and health institutions were processed before starting data collection. During data collection, study participants were asked for consent and were signed written agreements on consent forms. They were informed to interrupt the interview on desire. To ensure confidentiality, names were not used, instead code numbers were assigned to depict the results and the questionnaires were kept locked.

## Results

A total of 1730 women were interviewed. For analysis, 1668 women were considered because some 62 copies of questionnaire were excluded during the data cleaning process due to incompleteness and inconsistent values. Majority of the study subjects was Orthodox Christians (93.7%), Amhara by ethnicity (92.7%) and currently married (92.7%). At the time of their last birth, about 73.3% were between 20–34 years, 5.5% were teenagers (less than 20 years) and 21.2% were elderly gravid (35 years and above). The mean age was 28.2 ± 6.6 years, with a range of 16–51 years. The majority of the mothers (71%) and their husbands (63.4%) had no formal education (Table 2).

**Table 2:** Utilization of skilled maternal health services using selected individual level characteristics, North Gondar, 2012

**Characteristics**		**n**	**Number (percent) of women reporting skilled provider for:**		
			**ANC**	**Delivery care**	**PNC**
Birth order	1	335	132 (39.4)	77 (23.0)	31 (9.3)
	2-3	657	215 (32.7)	85 (12.9)	47 (7.2)
	4-5	439	116 (26.4)	43 (9.8)	17 (3.9)
	6+	237	75 (31.6)	26 (11.0)	10 (4.2)
Mother's education	No education	1183	339 (28.7)	129 (10.9)	56 (4.7)
	Primary	327	117 (35.8)	49 (15.0)	24 (7.3)
	Secondary and above	158	82 (51.9)	53 (33.5)	25 (15.8)
Husband education	No education	1057	286 (27.1)	116 (11.0)	50 (4.7)
	Primary	430	172 (40.0)	62 (14.4)	33 (7.7)
	Secondary and above	181	80 (44.2	53 (29.3)	22 (12.2)
Wealth quintile	Lowest	331	81 (24.5)	37 (11.2)	13(3.9)
	Second	340	107 (31.5)	43 (12.6)	15(4.4)
	Middle	306	112 (36.6)	44 (14.4)	30(9.8)
	Fourth	357	128 (35.9)	60 (16.8)	24(6.7)
	Highest	334	110 (32.9)	47 (14.1)	23(6.9)
Women have awareness on risk of pregnancy	No	522	90 (17.2)	52 (10.0)	13 (2.5)
	Yes	1146	448 (39.1)	179 (15.6)	92 (8.0)
Women preferred health professionals for the care	No	713	142 (19.9)	40 (5.6)	15 (2.1)
	Yes	955	396 (41.5)	191 (20.0)	90 (9.4)
Women have awareness on places to get skilled providers	No	341	45 (13.2)	14 (4.1)	7 (2.1)
	Yes	1327	493 (37.2)	217(16.4)	98 (7.4)
Pregnancy wontedness	Wanted/mistimed	1511	495 (32.8)	214 (14.2)	96 (6.4)
	Unwanted	157	43 (27.4)	17 (10.8)	9 ( 5.7)
ANC in previous pregnancy	No	957	200 (20.9)	107 (11.2)	42 (4.4)
	Yes	711	338 (47.5)	124 (17.4)	63 (8.9)
Women agree on planning health institutions as delivery place	No	432		38 (8.8)	
	Yes	1236		193 (15.6)	
ANC for this pregnancy by a skilled provider	No	1130		103 (9.1)	56 (5.0)
	Yes	538		128 (23.8)	49 (9.1)
Women agree to have PNC after normal delivery	No	767			26 (3.4)
	Yes	901			79 (8.8)
	Total	1668	538 (32.3)	231 (13.8)	105 (6.3)

About 32.3% (95% CI, 30.0%, 34.5%), 13.8% (95% CI, 12.2%, 15.5%) and 6.3% (95% CI, 5.1% to 7.5%) of women used antenatal, delivery and postnatal care by skilled providers, respectively. The bivariate analysis indicated that use of all indicators of skilled maternal services appeared to increase significantly with educational status of women and their husbands. Moreover, higher wealth quintiles, awareness of risk of pregnancy, preference for skilled provider, awareness about health facilities to get skilled professionals and previous experience of antenatal care showed a significant and positive association while higher birth order, and unwanted pregnancy showed a negative association in all maternal service indicators (Table 2).

The multilevel analysis was started from the intercept-only model to test the null hypothesis that there is no variation in maternal health services utilization between clusters (kebeles) and to decide in evaluation of the random effects at the community level. The results presented in Table 3 indicated that considerable heterogeneity between kebeles was observed for each indicator of skilled maternal care utilization. In all the three indicators, use of service is clustered significantly by kebele. The intra-class correlation in the empty model for antenatal, delivery and postnatal care by skilled provider indicated that 31%, 12%, and 25% of the total variance in use of the services was attributable to the differences across kebeles (Table 3).

**Table 3:** Parameter coefficients of the intercept-only model in using skilled maternal care, North Gondar, 2012

	**ANC by skilled provider**	**Birth attendance by skilled provider**	**PNC by skilled provider**
Random effects			
Level 2 variance	1.52* (0.63, 3.71)	0.45* (0.17, 1.18)	1.09* (0.35,3.38)
Rho - Intra-class correlation	0.31	0.12	0.25
Deviance	1832	1282	735

*Significant.

### Antenatal care by a skilled provider

Maternal education, preference for skilled provider, awareness about health facilities to get skilled professionals and previous experience of antenatal care were strong individual level predictors of the recent antenatal care by a skilled provider. The predictor ‘birth order’ indicates that women tend to use antenatal care by a skilled provider if their birth is their first time, and a small but significant difference was observed compared with those women with four or five births. The odds ratio indicates that use of antenatal services initially decreases with birth order up to the third category and increases thereafter.

Antenatal care by a skilled provider increases steadily with education so that women with secondary and above education were 68% more likely to use service as their counterparts with no education. Women who have had at least one antenatal care in previous pregnancies were about three times more likely to use skilled antenatal care for their recent pregnancy. Similarly, women who preferred health professionals for their maternity care and have awareness about health facilities to get skilled professionals significantly use skilled antenatal care compared with their counterparts.

The study further showed that communal level variables included in the model were not significant predictors of skilled antenatal services utilization.

Finally, a substantial proportion of the between kebele variance was explained by this model given the between kebele variation has been reduced from 1.52 to 0.76, a 50% reduction in unexplained variance between kebele ANC utilization. However, community level random effects are significant; the residual intra-class correlation is still appreciably large, indicating that even after controlling for individual, communal (kebele) level factors, there is still a considerable clustering of antenatal service utilization at the community (kebele) level. The between kebele variance of slopes indicated that the two variables, ANC in the previous pregnancies and preference for skilled providers, significantly varied across kebeles. That means, the relationships between these variables and ANC use by women were different for different kebeles (dependent on the proportion of ANC users in the kebeles) (Table 4).

**Table 4:** Results of the multilevel analysis by predictors of skilled maternity care, North Gondar, 2012

**Predictors**		**n**	**Adjusted odds ratio (95% CI)**		
**Level-1predictor variables (Individual and household characteristics)**			**ANC**	**Delivery care**	**PNC**
Birth order	1	335	1	1	1
	2-3	657	0.75 (0.53, 1.07)	0.60 (0.40, 0.90)	0.98 (0.56, 1.71)
	4-5	439	0.66 (0.44, 0.99)	0.51 (0.32, 0.85)	0.58 (0.28, 1.20)
	6+	237	0.79 (0.50, 1.25)	0.55 (0.31, 0.96)	0.60 (0.26,1.38)
Mother's education	No education	1183	1	1	1
	Primary	327	1.09 (0.80, 1.50)	1.17 (0.79, 1.72)	1.31 (0.76, 2.24)
	Secondary and above	158	1.68 (1.08, 2.60)	1.94 (1.21, 3.10)	1.72 (0.86, 3.47)
Husband education	No education	1057	1	1	1
	Primary	430	1.34 (0.98, 1.82)	0.83 (0.56, 1.22)	0.83 (0.49, 1.39)
	Secondary and above	181	1.22 (0.77, 1.94)	1.27 (0.77, 2.11)	0.85 (0.43, 1.70)
Wealth quintile	Lowest	331	1	1	1
	Second	340	1.02 (0.69,1.51)	1.08 (0.66, 1.78)	0.97 (0.44, 2.14)
	Middle	306	1.14 (0.75, 1.72)	1.24 (0.74, 2.08)	2.08 (1.01, 4.28)
	Fourth	357	1.03 (0.68, 1.56)	1.51 (0.92, 2.46)	1.35 (0.63, 2.86)
	Highest	334	0.83 (0.54, 1.27)	1.21 (0.71, 2.05)	1.44 (0.67, 3.13)
Awareness on risk of pregnancy	No	522	1	1	1
	Yes	1146	1.35 (0.96, 1.89)	1.10 (0.82, 1.75)	1.52 (0.79, 2.93)
Health professionals preferred for the care	No	713	1	1	1
	Yes	955	1.64 (1.14, 2.36)	2.29 (1.50, 3.51)	2.45 (1.31, 4.60)
Awareness on places to get skilled provider	No	341	1	1	1
	Yes	1327	1.63 (1.07, 2.49)	2.83 (1.54, 5.20)	1.48 (0.64, 3.42)
Pregnancy wontedness	Wanted/mistimed	1511	1	1	1
	Unwanted	157	0.79 (0.51, 1.22)	0.59 (0.34, 1.03)	0.67 (0.31, 1.42)
ANC in previous pregnancy	No	957	1	1	1
	Yes	711	3.39 (1.98, 5.80)	1.80 (1.31, 2.49)	1.92 (1.20, 3.08)
Skilled ANC for this pregnancy	No	1130		1	1
	Yes	538		1.90 (1.37, 2.63)	0.76 (0.48, 1.20)
**Level 2 predictor variables**					
**Communal (kebele) characteristics**					
Main source of income	Farming	1214	1	1	1
	Mixed	454	1.35 (0.47, 3.88)	1.64 (1.18, 2.28)	1.54 (1.03, 2.30)
Self sustained	No	445	1	1	1
	Yes	1223	1.29 (0.21, 8.09)		
Average distance to the nearest health center			0.83 (0.46, 1.48)	0.84 (0.69, 1.03)	0.80 (0.63, 1.02)
**Health facility characteristics**					
Signal functions	One or more missed	583	1	1	1
	All present	1085	1.14 (0.24, 5.51)	1.96 (1.13, 3.40)	1.16 (0.58, 2.35)
Availability of obstetric guidelines	No	900			1
	Yes	768			1.13 (0.59, 2.18)
Payment requirements during delivery	No	1252		1	
	Yes	416		0.53 (0.32, 0.88)	
Overall infrastructure	Not satisfactory	1153	1	1	1
	Satisfactory	515	1.64 (0.39,6.92)	0.98 (0.64, 1.51)	1.51(0.74, 3.10)
Basic equipments	Not satisfactory	878		1	
	Satisfactory	790		1.12 (0.73, 1.72)	
Random part					
Var (ANC in previous preg)			0.62 (0.22, 1.80)	-	-
Var (Preference of H. professionals for the care)			0.07 (0.14, 4.05)	-	-
Var(cons) i.e. level-2 variance			0.76 (0 .41, 2.21)	5.64e-19	2.13e-18
Rho - Intra-class correlation			0.19	Nearly 0	Nearly 0
Deviance (G^2^)			1652	1167	670

### Delivery care by a skilled provider

The significant individual level variables that predict use of delivery care by a skilled provider include; birth order, maternal education, preference for skilled provider, awareness about health facilities to get skilled professionals, experience of antenatal care for previous pregnancies and skilled antenatal care for the recent pregnancy. Women who had births for the first time were more likely to use skilled birth attendance compared with any of the birth order categories, and the difference was statistically significant in all comparisons. As we observed with the use of antenatal care, skilled attendance at birth increases significantly with secondary education. Women having at least one antenatal care in previous pregnancies or skilled antenatal care in their recent pregnancy were about two times more likely to have birth attendance by a skilled provider. Similar to the antenatal care service, women who preferred health professionals for their maternity care and have awareness about health facilities to get skilled professionals used significantly more delivery care by a skilled provider compared with their counter parts.

Three predictor variables from the second level, that is, source of income, signal function and payment requirements during delivery were significantly associated with skilled attendance at birth. Women who belonged to communities with mixed (farming and trading) source of income used skilled attendance 64% higher than those who belonged to only farming as the main source of income. The presence of all the six signal functions in the nearby basic essential obstetric care facility (health center) increased skilled attendance rate about two times compared to those health centers with at least one function is missing. By contrast, presence of payment requirements during delivery reduced utilization of skilled attendance by 47%.

The random part of this model indicated that almost all of the between kebele variance was explained by the included variables in the model. The model fitness was also improved significantly (Table 4).

### Postnatal care by skilled attendant

Three predictor variables, two from the individual level and one from the communal level significantly associated with postnatal care by a skilled provider. At the individual level, women who preferred skilled provider for their maternity care, and women who had experience of at least one antenatal care for their previous pregnancies used a skilled postnatal care more likely compared with those who did not have such characters.

At the communal level, the odds of postnatal care by a skilled provider significantly increased among women belonging to communities who had mixed (farming and trading) source of income compared with those belonging to only farming as the main source of income.

Like that of the skilled attendance at birth, the random part of the model indicated that almost all of the between kebele variance in the use of skilled postnatal care was explained by the variables in the model (Table 4).

## Discussion

Using a linked population and facility based survey and appropriate modeling framework, this study assessed the effect of individual, household, communal and health facility characteristics in utilization of antenatal, delivery, and postnatal care by a skilled provider. The results suggest that utilization of skilled maternal care by individual women is very low in the study area. The finding is consistent with the recent EDHS report and previous studies conducted in the surrounding area [5,19,20].

Our analysis indicates that utilization of skilled maternal care by individual women depends on the joint effect of individual, household, communal and facility characteristics. According to the intra-class correlation results, the contribution of unobserved communal level characteristics, were 31% for antenatal care, 12% for delivery care and 25% for postnatal care. In all the three intercept-only models, the contributions were significant and indicated that determining association without the control of variables at different levels would give a misleading result. This was also observed during analysis where many of the significant associations disappeared when the effect of clustering by kebele was controlled. Previous studies based on a similar analysis showed consistent findings [12,13].

At the individual level, variables related to awareness and perceptions were found to be much more relevant for skilled maternal service utilization. Women may get knowledge on the importance of skilled maternal care in different ways such as previous exposure to skilled maternal services, community based health educations, through community media or due to their better educational status. In this study, women with at least one antenatal care in previous pregnancies were significantly more likely, to use skilled maternal care for all the three indicators of their recent pregnancy. The positive effect of maternal service in the previous birth on current maternal care was observed in different studies done elsewhere [12,21,22].

Having awareness about health facilities to get skilled professionals was a significant predictor of both skilled antenatal and delivery care. The effect of awareness was also observed in community-based interventions focusing on awareness creation. For example, in Ethiopia, higher rate of household visits and awareness by health extension and community health workers were associated with improved antenatal care use and postnatal care visits [23]. Better educational status is believed to be an important factor for better awareness and positive attitude related to maternal health service utilization. As expected, skilled maternal care during pregnancy, delivery, and postnatal period increased steadily with education. Women with secondary and above education were also significantly associated with antenatal and delivery care by a skilled provider. Previous studies support our findings [13,19,24,25].

One of the expected effects of knowledge about an issue is change on individual attitudes. Women who have confidence on skilled providers and their care tend to use maternal health services by a skilled provider. In our analysis, significantly higher utilization rate of all skilled maternal health service indicators was observed among women who perceive that health professionals are better and safer (prefer skilled providers) for their maternity care. The implications of this finding is that community based health education about the benefit of skilled maternal care by targeting women who prefer non-skilled providers as well as improving the quality of care by providers will bring a positive contribution for utilizing skilled maternal care.

The predictor birth order indicates that women tend to use skilled maternal care if their birth is the first, and its significant effect is observed in antenatal and delivery care. The variations observed in the odds ratios can be related with the risk perception of women that varies overtime. Many women (48.3%) believe the first pregnancy is risky compared with the next consecutive pregnancies. However, as the number of pregnancies exceeds a certain limit, they start to think about another risk. Because of this, many women (41.3%) believe that having too many births is risky for a woman.

Women’s health seeking behavior is also influenced by the cost of maternity services and their capacity to cover the expected expenses. For instance, a substantial proportion of antenatal care users did not deliver or use postnatal care by a skilled provider. On the one hand, maternity services, especially delivery care, are expensive. Studies indicate that delivery care use among antenatal care users is highly correlated with wealth [26]. On the other hand, delivery occurs suddenly. As a result, women have home birth by non-skilled providers due to transportation problems and lack of preparation. The positive contribution of better wealth status for all maternity service indicators and its significant contribution to postnatal care are also observed in our analysis. The study further evaluated the contribution of wealth indicators at kebele level. Women living in kebeles with mixed (farming and trading) source of income were utilizing significantly more skilled delivery and postnatal care than those living on farming only. The finding indicates that the presence of different sources of income for covering payments of transportation and other services contributed to the existence of higher rate of expensive maternal services in the communities. In this study, payment requirement at the time of delivery was an important barrier to delivery service by a skilled provider. The negative effect of the cost of maternity services and the need for reducing it was also observed in a study conducted in Tanzania. In the Tanzania study, standard maternity services were costly. For most households, maternal health care could take more than half of their annual consumption. The study further indicated that by subsidizing maternity care, women, especially those in the lowest socio-economic category would experience the greatest increase in service utilization [27]. The findings of our study and previous evidences imply that for better achievements in the utilization of skilled maternal health services, there should be mechanisms to reduce or avoid the costs of maternal services.

Another strong facility level predictor for skilled maternal care utilization was the performance of health facilities. The presence of all the six signal functions in the nearby basic essential obstetric care facility (health center) positively contributed to the utilization of all indicators of skilled maternal services, and its effect was significant on skilled attendance rate. Functioning obstetric facility means performing the essential services for normal situations and complications and these services should be available 24 hours a day and 7 days a week. The presence of all signal functions reflects better performance (quality) of a health facility. Furthermore, the findings of this study imply that improving the quality of care and monitoring facilities to perform the expected function at their level will be one of the strategies to increase the utilization rates of maternity care by a skilled provider.

### Limitations

One of the limitations of this study was that women faced difficulties to differentiate the type of skilled providers during interview. To reduce the problem, data collectors gave further clarifications by collecting information on types of providers (doctor, health officer, nurse, midwife, health extension worker or others) from health institutions that gave services. Errors may occur in knowing the categories of health professionals. However, there were no serious difficulties in categorizing service providers as skilled or non-skilled, so it did not affect the interpretation of results. The potential limitations expected during analysis of determinant factors were minimized.

## Conclusions

Factors operating at individual and communal (kebele) levels play a significant role in determining the utilization of skilled maternal health services, so controlling unexplained variations of the higher level is very important for preventing misleading associations. At the individual level, health-seeking behavior of a woman was more dependent on her awareness and perception on skilled provider or care. Included communal factors were relevant for both delivery and PNC. Health facility characteristics, including performance (functionality) and cost of service were more important for use of skilled delivery care than other maternity services. Improving community awareness and perception on skilled providers and their care by targeting women who prefer non-skilled providers and those who do not have any awareness is very important. Safe motherhood education using the available communication networks in the rural communities (health development army) and innovative informational campaigns are strategies to improve the intended awareness and perceptions of women. Furthermore, ensuring the seamless performance of basic essential obstetric care facilities (health centers) is also very critical, especially for improving the rate of skilled attendance at birth.

## Competing interests

The authors declare that they have no competing interests.

## Pre-publication history

The pre-publication history for this paper can be accessed here:

http://www.biomedcentral.com/1472-698X/13/20/prepub
